# Hypoxic Stress Decreases c-Myc Protein Stability in Cardiac Progenitor Cells Inducing Quiescence and Compromising Their Proliferative and Vasculogenic Potential

**DOI:** 10.1038/s41598-017-09813-x

**Published:** 2017-08-29

**Authors:** Michael A. Bellio, Mariana T. Pinto, Victoria Florea, Paola A. Barrios, Christy N. Taylor, Ariel B. Brown, Courtney Lamondin, Joshua M. Hare, Ivonne H. Schulman, Claudia O. Rodrigues

**Affiliations:** 1 Interdisciplinary Stem Cell Institute, University of Miami Miller School of Medicine, Miami, Florida United States of America; 2 Department of Medicine, Cardiovascular Division, University of Miami Miller School of Medicine, Miami, Florida United States of America; 3 Department of Medicine, Katz Family Division of Nephrology and Hypertension, University of Miami Miller School of Medicine, Miami, Florida United States of America; 4 Department of Molecular and Cellular Pharmacology, University of Miami Miller School of Medicine, Miami, Florida United States of America

## Abstract

Cardiac progenitor cells (CPCs) have been shown to promote cardiac regeneration and improve heart function. However, evidence suggests that their regenerative capacity may be limited in conditions of severe hypoxia. Elucidating the mechanisms involved in CPC protection against hypoxic stress is essential to maximize their cardioprotective and therapeutic potential. We investigated the effects of hypoxic stress on CPCs and found significant reduction in proliferation and impairment of vasculogenesis, which were associated with induction of quiescence, as indicated by accumulation of cells in the G0-phase of the cell cycle and growth recovery when cells were returned to normoxia. Induction of quiescence was associated with a decrease in the expression of c-Myc through mechanisms involving protein degradation and upregulation of p21. Inhibition of c-Myc mimicked the effects of severe hypoxia on CPC proliferation, also triggering quiescence. Surprisingly, these effects did not involve changes in p21 expression, indicating that other hypoxia-activated factors may induce p21 in CPCs. Our results suggest that hypoxic stress compromises CPC function by inducing quiescence in part through downregulation of c-Myc. In addition, we found that c-Myc is required to preserve CPC growth, suggesting that modulation of pathways downstream of it may re-activate CPC regenerative potential under ischemic conditions.

## Introduction

Heart failure is the leading cause of death from cardiovascular disease and the end result of a number of chronic illnesses, including hypertension, diabetes, and coronary artery disease^1^. It has been estimated that approximately 1 in every 4 deaths in the United States is due to heart failure associated with ischemic heart disease^2^. Long-term survival after heart failure diagnosis remains poor despite recent improvements in pharmacological and surgical therapy^3–7^. In the last decade, multiple studies have suggested a primary role for myocyte loss in the induction and progression of heart failure^8–13^. Development of therapeutic approaches to regenerate and/or protect the heart through improved cell survival and production of functional myocytes may alleviate heart failure and reduce the number of deaths associated with ischemic heart disease.

Stem cell-mediated therapy to treat heart failure has been pursued as a potential alternative to current approaches to preserve myocardial function and prevent cardiac remodeling^3, 4^. However, multiple clinical trials have shown mixed results and modest impact on cardiac function^14–20^. The discovery of cardiac progenitor cells (CPCs) capable of promoting myocardial regeneration in experimental models, suggested a new therapeutic venue involving direct stimulation of endogenous repair mechanisms in the heart^21–30^. Although CPC potential to replace myocytes lost after ischemic injury has been shown to be limited, the precise role played by CPCs in response to ischemic injury is largely unknown and optimization of CPC survival and function during myocardial infarction may be critical^31, 32^. Oxygen delivery through the vascular network is critical to prevent myocyte loss. Despite their vasculogenic properties, resident CPCs are not able to promote sufficient revascularization after ischemic injury, suggesting that the hypoxic environment may impair their regenerative potential. To date, few studies have investigated the factors and signaling pathways that regulate CPC survival, regeneration and differentiation in the setting of ischemic disease^33^. An essential goal for research in this area will be to elucidate the effectors and modulators of CPC stress susceptibility in order to maximize cardioprotective and stem cell therapeutic strategies.

The transcription factor c-Myc is well known for its role in the regulation of proliferation, differentiation and survival of many cell types^34, 35^. Recent studies have shown that c-Myc may be essential to preserve self-renewal and pluripotency of adult hematopoietic and embryonic stem cells^36–39^. The role of c-Myc in driving stemness triggered a series of studies on its potential targeting in cancer stem cells^40, 41^. In addition, c-Myc has been employed as an important tool in reprogramming during cell-mediated therapy to treat several conditions, including heart failure^42^. Here we report a novel role for c-Myc in controlling adult CPC cell fate decisions in response to stress. Our results suggest that downregulation of c-Myc under ischemic injury may contribute to impairment of CPCs regenerative potential.

## Materials and Methods

### Cell Lines and Culture Conditions

Cardiac progenitor cells (CPCs) were isolated from mouse myocardium by magnetic cell sorting with anti-c-Kit antibody as previously described^43^. Cell identity was confirmed by flow cytometry analysis^44^. CPCs were maintained in HAM’S F12 media (Lonza) supplemented with 10% fetal bovine serum (FBS), 10 ng/mL mouse basic fibroblast growth factor (bFGF), 20 ng/mL mouse epidermal growth factor (EGF), 10 ng/mL mouse leukemia inhibitory factor (LIF), 1% insulin-transferrin-selenite supplement (ITS) and 1% penicillin/streptomycin. All cells were cultured under 21% O_2_ and 5% CO_2_ atmosphere, at 37 °C. Ischemic hypoxia treatment consisted of incubation of cells under 0.5% O_2_ in a temperature and humidity controlled chamber (Coy Laboratory).

### Cell Proliferation Assays

The effect of hypoxia on CPC proliferation was assessed by analyzing growth rates for 4–6 days (96–144 hours). Cells were plated at an initial density of 1 × 10^5^ cells in 60 mm culture dishes, allowed to attach and placed under normoxia (21% O2) and hypoxia (0.5% O_2_) conditions the same day. The number of cells was computed daily using an automated cell counter (Bio-Rad Laboratories). For quiescence experiments, cells were cultured for 3 days (72 hours) in hypoxia and then transferred to normoxia for 3 more days. Cell numbers were computed every 3 days. Population doubling was estimated using a calculator tool available on line (http://doubling-time.com/). The effect of c-Myc inhibition on CPC growth was determined by staining cells with 1 μM of CellTrace Far Red Cell Proliferation Dye (Thermo Fisher Scientific) followed by treatment with 40 μM the c-Myc inhibitor 10058-F4 (Sigma-Aldrich, #F3680) or vehicle control (DMSO). After a period of 3 days, cells were harvested and analyzed by flow cytometry for changes in mean fluorescence intensity (dye dilution is an indication of proliferation) using a BD LSR II flow cytometer (BD Biosciences) and analyzed with FloJo software. At least 10,000 events were computed per sample. To determine the involvement of GSK-3β in CPCs proliferation and c-Myc expression, growth curves were performed in the presence of the inhibitor TCS2002 (Tocris Bioscience, #3869) in doses ranging from 50–100 nM and DMSO vehicle control.

### Cell Cycle Analysis

Progression through the cell cycle was assessed using a combination of Ki67 and 7-AAD DNA stain^45–47^. Briefly, CPCs were harvested by trypsinization after hypoxia and c-Myc inhibitor treatments performed essentially as described above, immediately fixed in 70% ethanol and stored at −20 °C until use. Aliquots of fixed cells containing at least 1 × 10^5^ cells were transferred to test tubes. Cells were pelleted down and washed twice with staining buffer (1% FBS in PBS) followed by resuspension in 100 ul of staining solution containing 5 μl of Alexa Fluor 488-conjugated anti-Ki67 antibody (BD Pharmingen, #561165) or isotype control (BD Pharmingen, #565572). Cells were incubated in antibody solution for 30–45 minutes at room temperature, washed in staining buffer and resuspended again in a second staining solution containing 5 μl of 7-AAD (BD Biosciences, #559925) for 10 minutes, also at room temperature. Ki67 staining was analyzed by flow cytometry alone and in combination with 7-AAD (double stained cells) for assessment of proliferation and cell cycle progression^45–47^, respectively. Data was collected using the BD LSR II flow cytometer (BD Biosciences) and analyzed with FlowJo software. At least 10,000 events were computed per sample.

### Tube Formation Assay

The vasculogenic potential of CPCs grown under normoxia and hypoxia for 3 days (72 hours) was analyzed using a tube formation assay on Matrigel (BD Biosciences, #354234). Cells were harvested by brief trypsinization and plated on 24-well culture dish (5–6 × 10^4^ cells/well) pre-coated with 300 μl of regular growth factor Matrigel in endothelial basal media (Lonza) supplemented with 0.1% bovine serum albumin. After a period of 6 and 24 hours, samples were analyzed for tube formation potential. At least 6 images were collected at 4–10X magnification from random fields for each sample. Calcein-AM (Thermofisher Scientific, #L3224) was used in the last time point to determine cell viability.

### Quantitative Detection of Senescence-Associated-β-Galactosidase Activity

Senescence associated-β-galactosidase activity was quantitatively determined in CPC lysates obtained from cells cultured under normoxia and hypoxia from 24–96 hours. Total protein concentration was determined by the method of Bradford (Bio-Rad Laboratories, #5000001 ). Samples were harvested and processed according to manufacturer’s instructions (Cell Biolabs, #CBA-231) using 10 μg of protein per assay. Activity was determined by fluorescence detection using a SpectraMax M5 plate reader (Molecular Devices). Results are expressed as fluorescence units.

### Cell Death Analysis

Cell death was determined using Live/Dead staining and detection of lactate dehydrogenase activity. Live and dead cells were assessed in CPCs cultured under normoxia and 0.5% hypoxia every day for a total period of 4 days (96 hours) using Calcein-AM and Ethidium homodimer-1 (Thermo Fisher Scientific, #L3224) according to manufacturer’s instructions. A positive control was generated by treating cells with 200 μM hydrogen peroxide. Lactate Dehydrogenase Activity (LDH), which is released in culture supernatants due to an increase in permeability during cell death, was determined in culture supernatants using a Cytotoxicity Detection Kit (LDH) (Roche, #11644793001). CPCs were plated in 96-well plates in triplicate (1 × 10^3^ cells/well) using phenol-red free culture media and allowed to grow under normoxia (21% O_2_) and hypoxia (0.5% O_2_) for 72 hours. After this time, LDH activity was measured *in situ*, according to manufacturer’s instructions. Results were normalized to the number of cells to compensate for difference in growth and activity expressed as absorbance units (AU).

### RNA Isolation and Gene Expression Profiling by Quantitative RT-PCR

RNA was extracted from CPCs grown under normoxia and hypoxia using RNeasy kit (Qiagen, #74104) according to manufacturer’s instruction. All samples were subjected to in-column DNAse treatment (Qiagen, #79254) prior to cDNA synthesis. First strand cDNA was prepared using High Capacity cDNA Reverse Transcriptase kit (Thermo Fisher Scientific, #4368814). Reverse-transcribed cDNA was used for Quantitative RT-PCR using TaqMan probes for mouse c-Myc (Mm00487804_m1), Flk-1 (Mm01222419_m1), Vegf (Mm01281449_m1) and p21 (Mm04205640_g1) (Applied Biosystems, Life technologies). Fold-changes in hypoxia samples were calculated relative to normoxia control by the ΔΔCt method using at least 2 endogenous controls for normalization.

### Protein Stability Assays

CPCs were incubated under normoxia and hypoxia for 48 hours and treated with 5 μg/ml of the protein synthesis inhibitor cycloheximide (Sigma-Aldrich, #F3680). Protein lysates were collected from untreated normoxia and hypoxia controls (baseline) and from cycloheximide-treated samples every 10 minutes for a total period of 1 hour. To further confirm that hypoxia affected c-Myc protein degradation rate, samples cultured under hypoxia for 48 hours were also treated with 10 μg/ml of the proteasome inhibitor MG132 (Sigma-Aldrich, #M7449). Lysates were collected at 4 and 24 hours after treatment. In both cases samples were analyzed by western blot as described below.

### Protein Expression Analysis by Western Blot

Protein lysates were prepared in RIPA buffer and quantified using Bradford Assay (Bio-Rad,﻿ #5000001). SDS-PAGE and Western blots were performed according to standard procedures using antibodies against the cell cycle regulators c-Myc (Cell Signaling, #5605), p21 (Santa Cruz Biotechnology, #SC-756) and Sirt1 (Abcam, #ab110304); the differentiation markers CDH5 (Cell Signaling, #2158 S), PECAM1 (Cell Signaling, #3528 S), α-SMA (Sigma, #A2547) and SM22-α (Abcam, #ab14106), the kinases GSK-3β (Cell Signaling, #9832 S), AKT (Cell Signaling, #9272) and phosphorylated forms phospho-GSK-3β (Ser9) (Cell Signaling, #5558 S) and phospho-AKT (Ser473) (Cell Signaling, #9271), and endogenous controls Actin (Sigma-Aldrich, #A2066), Gapdh (Cell Signaling, #5174) and Tubulin (Cell Signaling, #2146). Prior to blocking and antibody incubation, membranes were stained with Ponceau S reagent (Sigma-Aldrich, #P3504) to visualize protein bands and cut at specific sizes so different antibodies could be tested at the same time. Densitometry analysis of western blots was performed using Quantity One software (Bio-Rad Laboratories).

### Statistical Analysis

Data obtained from all experiments were analyzed for significance using Student t-test or one-way analysis of variance (ANOVA) followed by software-recommended post-tests, depending on sample distribution, using Microsoft Excel, Sigma-Plot and GraphPad Prism Software. For a given parameter, p < 0.05 was considered significant. All data are presented as means ± standard error.

## Results

### Hypoxic stress reduces CPC proliferation

**Figure 1:**
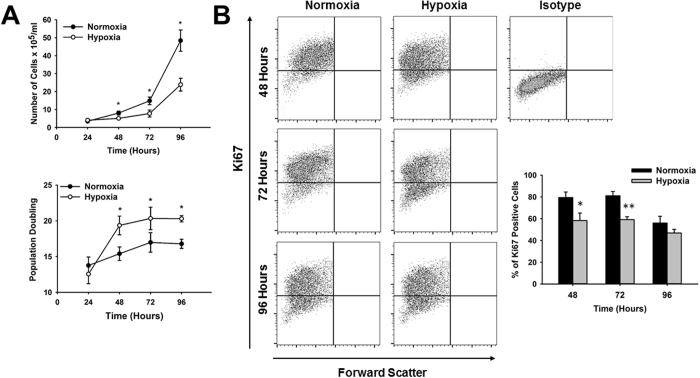
Hypoxic stress reduces cardiac progenitor cell proliferation. **(A)** Time-course analysis of CPC growth represented by number of cells (top panel) and population doubling (lower panel) under normoxia (closed circles) and hypoxia (open circles) over a total period of 96 hours (n = 7, *p < 0.05). **(B)** Quantitative analysis of Ki67 positive cells after exposure of CPCs to hypoxia by fluorescence activated cell sorting. Representative images under normoxia and hypoxia from 48–96 hours are shown. Graph indicates the average percentage of Ki67 positive cells under normoxia (black bars) and hypoxia (gray bars) at different time points (n = 3, *p < 0.05, ** < 0.005).

Self-renewal is essential for expansion of resident progenitor cells in response to injury and efficient tissue repair. We investigated the effect of hypoxic stress on CPC proliferation. Cells were cultured under ischemic hypoxic (0.5% O_2_) and normoxic (21% O_2_) conditions, and proliferation estimated by counting cell numbers daily over a period of 96 hours. Proliferation of CPCs gradually declined under hypoxia in a time-dependent manner relative to normoxia-control on average by 43.31 ± 4.32% (34.70 ± 5.96% at 48 hours, 47.25 ± 10.35% at 72 hours and 48.00 ± 9.42% at 96 hours). Significant reduction in cell numbers was observed as early as 48 hours under hypoxia relative to normoxia (5.08 ± 0.43 × 10^5^ vs 8.04 ± 1.03 × 10^5^ cells/ml, p < 0.03, n = 5) (Fig. 1A, upper panel). This effect was specific to hypoxic stress as no difference in cell proliferation was observed in cells grown in 5% O_2_ (Supplementary Figure 1). The decrease in cell numbers observed under hypoxia was associated with an average increase in doubling time of 23.13 ± 1.55% (26.17 ± 6.32% at 48 hours, 21.34 ± 7.95% at 72 hours and 22.09 ± 6.73% at 96 hours) relative to cells grown under normoxia. The increase in doubling time was evident at 48 hours relative to normoxia (19.36 ± 1.31 vs. 15.40 ± 0.95 population doubling, p < 0.02, n = 5) and remained constant at 72 (20.34 ± 1.56 vs. 16.99 ± 1.37 population doubling, p < 0.04, n = 6) and 96 hours (20.30 ± 0.44 vs. 16.79 ± 0.64 population doubling, p < 0.02, n = 6) (Fig. 1A, lower panel).

Expression analysis of the cell cycle marker Ki67 showed that proliferation was reduced under hypoxia by 26.91 ± 5.18% (58.30 ± 6.86 vs. 79.43 ± 5.14% of Ki67 positive cells, p < 0.04, n = 3) at 48 hours and 26.95 ± 1.99% (59.20 ± 2.61 vs. 81.13 ± 3.87, p < 0.005, n = 3) at 72 hours relative to normoxia. At 96 hours, reduction in Ki67 expression in hypoxia was not significant, which is likely due to contact inhibition as normoxia cultures reach confluence earlier than those grown under hypoxia (Fig. 1B).

### Hypoxic stress does not induce CPC cell death or cellular senescence

**Figure 2:**
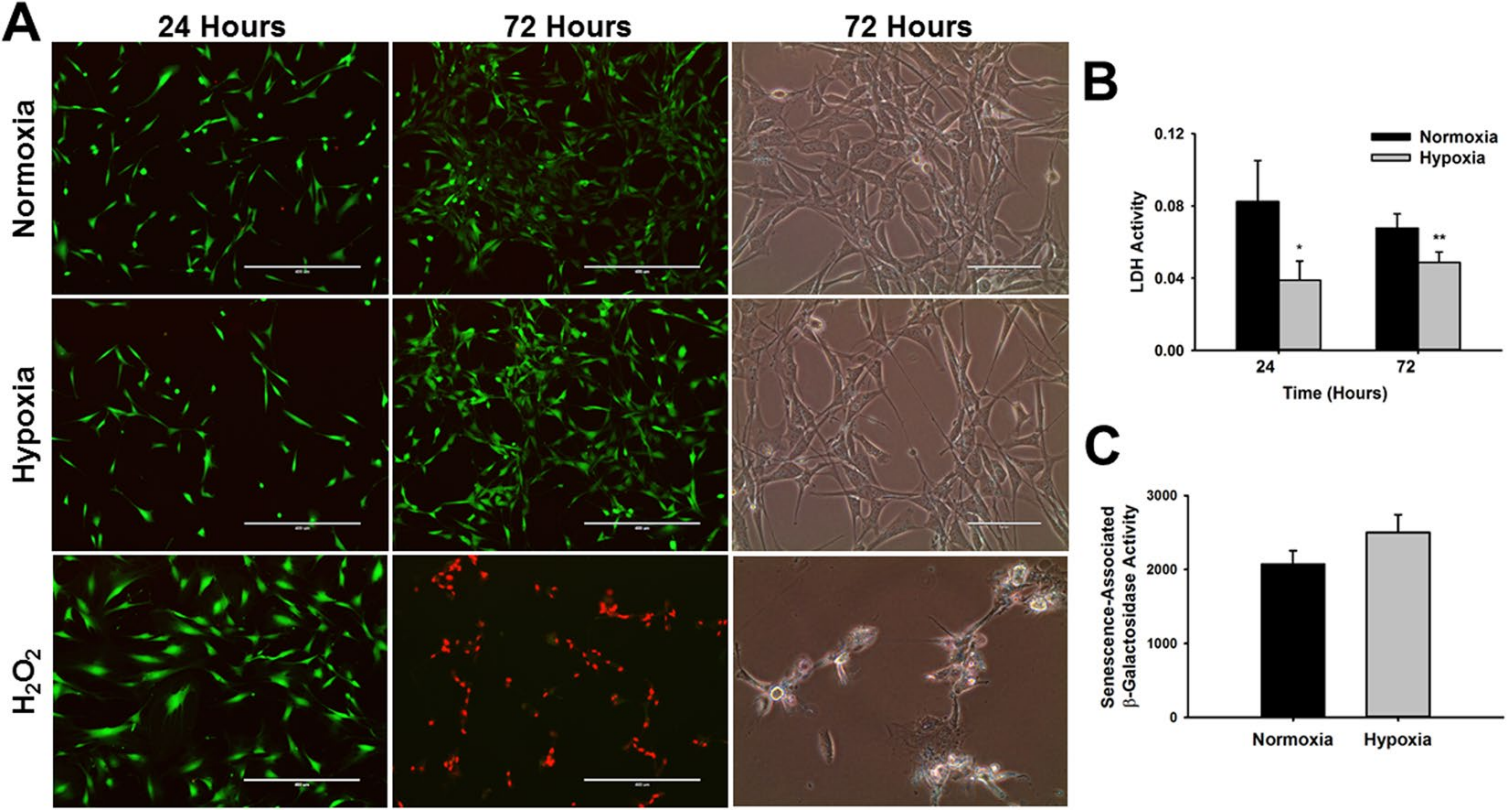
Hypoxia exposure does not induce cardiac progenitor cell death or senescence. (**A**) Representative images of CPCs stained with the live/dead dyes calcein AM (green) and ethidium homodimer-1 (red) under normoxia and hypoxia at 24 and 72 hours (n = 3, 10X magnification). A positive control was generated by treatment of CPCs with 200 µM hydrogen peroxide (H_2_O_2_) during the same time-frame and bright field images are shown for analysis of morphological changes after hypoxia and hydrogen peroxide treatments relative to control. (**B**) Detection of lactate dehydrogenase activity in supernatants of CPCs cultured under normoxia (black bars) and hypoxia (gray bars) at 24 and 72 hours (n = 6, *p < 0.05, **p < 0.005). **(C)** Detection of senescence associated-β-galactosidase activity in cell lysates of CPCs cultured under normoxia (black bars) and hypoxia (gray bars) for 72 hours (n = 5).

Most cell types respond to stress by undergoing apoptosis or senescence. In order to confirm that reduction in CPC numbers under hypoxia was not associated with cell death we used two different approaches. We performed a live/dead assay with ethidium homodimer-1 and calcein-AM during the course of growth under normoxia and hypoxia. In this assay, cell death is indicated by loss of calcein-AM staining (green) and uptake of ethidium homodimer-1 (red). Representative images of cells in culture at 24 and 72 hours under each experimental condition are shown in Fig. 2A. A positive control was generated for comparison purposes by treating CPCs with hydrogen peroxide. Results show that the number of cells labeled with calcein-AM is reduced in hypoxia relative to normoxia at 72 hours as expected. However, this was not associated with cell death as indicated by the lack of ethidium homodimer-1 staining (red) and preservation of the calcein-AM stain (green). In contrast, hydrogen peroxide-treated cells die with time, as indicated by loss of calcein-AM stain and uptake of ethidium homodimer-1 (H_2_O_2_, Fig. 2A lower panel). Hydrogen peroxide-treated cells also undergo significant morphological changes associated with death, while cells grown under normoxia and hypoxia maintain their normal morphological features (Fig. 2A, bright field images).

In addition to the live/cell dead assay described above, we also estimated cell death by detection of lactate dehydrogenase activity in culture supernatants, which is expected to leak from dying cells. Results show that there were no significant changes in activity with time in culture (72 hours relative to 24 hours) in normoxia (0.068 ± 0.01 vs. 0.083 ± 0.02 absorbance units, p < 0.45, n = 6) or hypoxia (0.049 ± 0.01 vs. 0.039 ± 0.01 absorbance units, p < 0.20, n = 6) (Fig. 2B), indicating that the hypoxia treatment is not triggering cell death with time. Interestingly, we found that LDH activity is significantly decreased under hypoxia relative to normoxia by 48.36 ± 6.40% at 24 hours (0.039 ± 0.01 vs 0.083 ± 0.023 absorbance units, p < 0.03, n = 6) and 27.06 ± 4.31% at 72 hours (0.049 ± 0.01 vs 0.068 ± 0.01 absorbance units, p < 0.004, n = 6).

We next investigated if one of the potential causes of reduced proliferation in CPCs was related to the development of senescence. Quantification of senescence-associated-β-galactosidase activity in CPCs grown under hypoxia relative to cells grown under normoxia did not show any significant difference between groups (Fig. 2C, n = 5).

### Hypoxic stress does not affect CPC differentiation but impairs vasculogenesis

**Figure 3:**
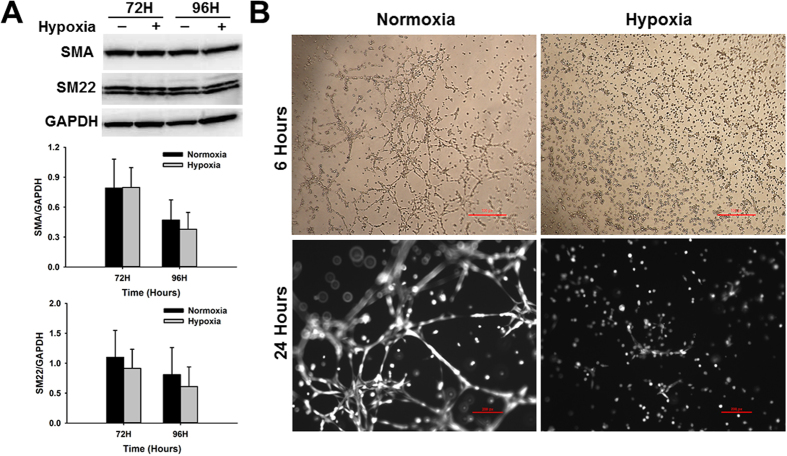
Hypoxic stress does not induce cardiac progenitor cell differentiation but impairs vasculogenic potential. (**A**) Representative western blot images showing the expression of smooth muscle markers in CPCs at 72 and 96 hours under normoxia (black bars) and hypoxia (gray bars). Graphs represent the expression of differentiation markers determined by densitometry analysis of all blots normalized to Gapdh endogenous control (n = 5). (**B**) Representative images of normoxia and hypoxia-treated CPCs at 6 hours (top panels, 4X magnification) and 24 hours (lower panel, 10X magnification) after culturing on Matrigel (n = 5). Cells in 24 hour-time point images were stained with calcein-AM to indicate that they were alive by the end of the study.

Reduction in cell proliferation may be a consequence of cellular differentiation. As previously reported, most mouse CPCs undergo differentiation into smooth muscle and endothelial lineages^43, 48^. To determine if the decline in CPC proliferation after exposure to hypoxic stress was due to an increase in cell differentiation, we analyzed the expression of endothelial and smooth muscle markers at 72 and 96 hours, after exposure to hypoxia. Our results show that hypoxia did not cause any significant changes in the expression of the smooth muscle markers α-SMA and SM22-α (Fig. 3A).

Analysis of CPC vasculogenic potential indicate that hypoxia severely impacted tube formation. Branching morphogenesis is an essential process required during vasculogenesis in which progenitor cells associated with the vasculature or located in the bone marrow are activated and migrate towards growth factor gradients to form new vascular structures. This process can be replicated *in vitro* by culturing cells in basement membrane matrix (Matrigel). We plated normoxia-control and hypoxia-treated CPCs on Matrigel and analyzed tube formation during a period of 24 hours. While normoxia control cells were able to form tubular structures as expected in as little as 6 hours after plating on Matrigel, hypoxia-treated cells remained dispersed in the matrix even after 24 hours (Fig. 3B, n = 5). Our results show that the vasculogenic potential of hypoxia-treated CPCs was impaired despite the presence of growth factors in the Matrigel matrix, suggesting that hypoxic stress affects CPC ability to respond to pro-angiogenic stimulation.

### Hypoxic stress induces CPC quiescence

**Figure 4:**
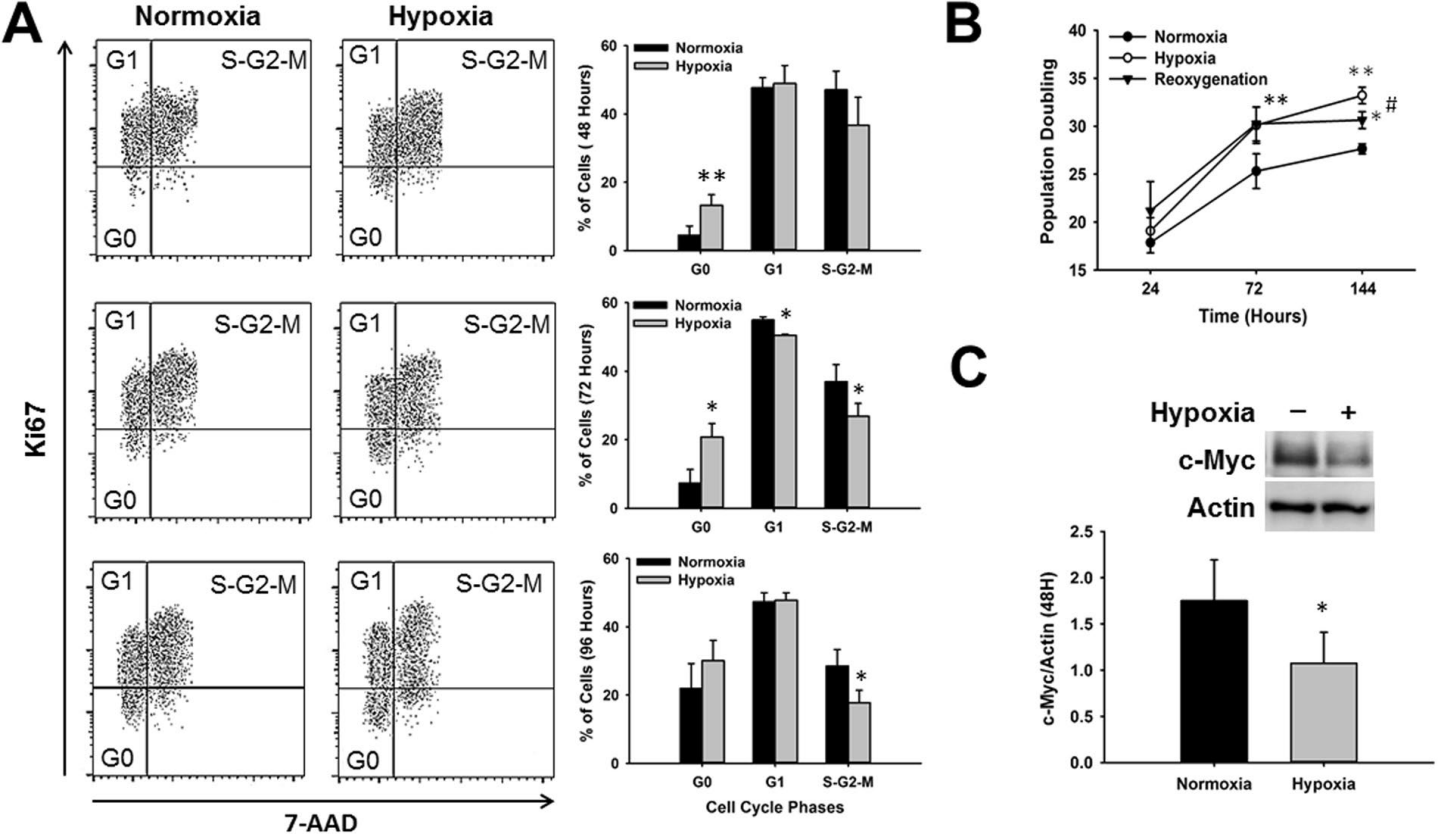
Hypoxic stress leads to cardiac progenitor cell quiescence and a decrease in the expression of c-Myc. **(A)** Time-course analysis of CPCs cell cycle progression under normoxia and hypoxia. Dot Plots show representative images of cells stained with 7-AAD and Ki67 and analyzed at 48 (top panel), 72 (middle panel) and 96 (lower panel)-hours. Graphs indicate the average percentage of cells in each phase of the cell cycle per time point under normoxia (black bars) and hypoxia (gray bars) (n = 3, *p < 0.05, **p < 0.005). **(B)** Time-course analysis of CPC growth represented by population doublings under normoxia (closed circle), hypoxia (open circles) and hypoxia followed by normoxia (reoxygenation, closed triangles) (n = 7, **p < 0.005 Hypoxia vs Normoxia, *p < 0.05 Reoxygenation vs Normoxia, ^#^p < 0.05 Reoxygenation vs Hypoxia). **(C)** Representative western blot image showing decrease in c-Myc protein expression at 48 hours. Graph represents c-Myc expression determined by densitometry analysis of all blots normalized to Actin endogenous control (n = 4, *p < 0.05).

Our results show that the reduced proliferation observed after exposure of CPCs to ischemic hypoxia was not associated with an increase in cell death, development of senescence or cell differentiation. Moreover, CPCs vasculogenic potential was impaired. One possible explanation for our findings is that ischemic hypoxia may lead CPCs into a quiescence state. In order to test this hypothesis we performed cell cycle analysis of CPCs cultured under normoxia and hypoxia. In all time points tested, the percentage of CPCs in G0-phase was higher in hypoxia than normoxia. However, results were significantly different only at 48 (13.28 ± 3.07 vs. 4.53 ± 2.68% of cells, p < 0.003, n = 3) and 72 hours (20.80 ± 3.92 vs. 7.36 ± 3.97% of cells, p < 0.01, n = 3). Although the percentage of hypoxia-treated cells in G0-phase was still higher than normoxia at 96 hours, results were not significant (30.10 ± 5.97 vs. 21.93 ± 7.32, p < 0.06, n = 3), as normoxia-treated cells also start to accumulate in G0 (Fig. 4A). This is probably a result of contact inhibition as CPCs reach confluence faster under normoxia than under hypoxia.

To further confirm if hypoxia was inducing CPC quiescence, we determined if the decrease in cell proliferation could be reversed when cells were placed back under normoxia after hypoxia exposure (reoxygenation). As expected, CPCs exposed to ischemic hypoxia showed an increase in doubling time of approximately 19.89 ± 4.61% relative to normoxia (30.10 ± 1.89 vs. 25.31 ± 1.81 population doubling, p < 0.002, n = 7). After CPCs were moved back to normoxic conditions for 3 days (144-hour time point), signs of significant growth recovery were observed relative to hypoxia as indicated by a decrease in doubling time (30.64 ± 0.87 vs. 33.20 ± 0.86 population doubling, p < 0.03, n = 7), in the direction of control levels (27.64 ± 0.53 population doubling, p < 0.03, n = 7). Altogether, our findings suggest that hypoxic stress induces CPC quiescence (Fig. 4B).

### Hypoxia-induced CPC functional decline is associated with reduced c-Myc expression

In order to identify the mechanism for CPCs functional decline under hypoxic stress, we next investigated the role of c-Myc, a transcriptional factor implicated in the cell fate decisions related to growth, proliferation, differentiation and survival, and an important mediator of stemness^36–39^. The expression of c-Myc is downregulated relative to normoxia control as early as 24 hours after CPC exposure to hypoxia, but significantly only after 48 hours (1.07 ± 0.33 vs 1.75 ± 0.44 c-Myc/Actin ratio, p < 0.02, n = 4) (Fig. 4C). Time-course analysis of c-Myc expression showed an average decline of 39.85 ± 2.43% under hypoxia. There was no significant difference in the percentage of decrease in c-Myc expression among time points (40.87 ± 7.83% at 48 hours vs. 35.24 ± 5.82% at 72 hours vs. 43.46 ± 7.95% at 96 hours) (Supplementary Figure 2), suggesting that once c-Myc expression is downregulated, the decrease is sustained with time. Comparison between c-Myc expression and cell numbers under hypoxia, relative to normoxia, shows a virtually identical rate of decline for both parameters with time. In fact, Pearson correlation analysis indicates a perfect fit (r = 0.993).

### c-Myc regulates CPC proliferation and cell cycle progression

**Figure 5:**
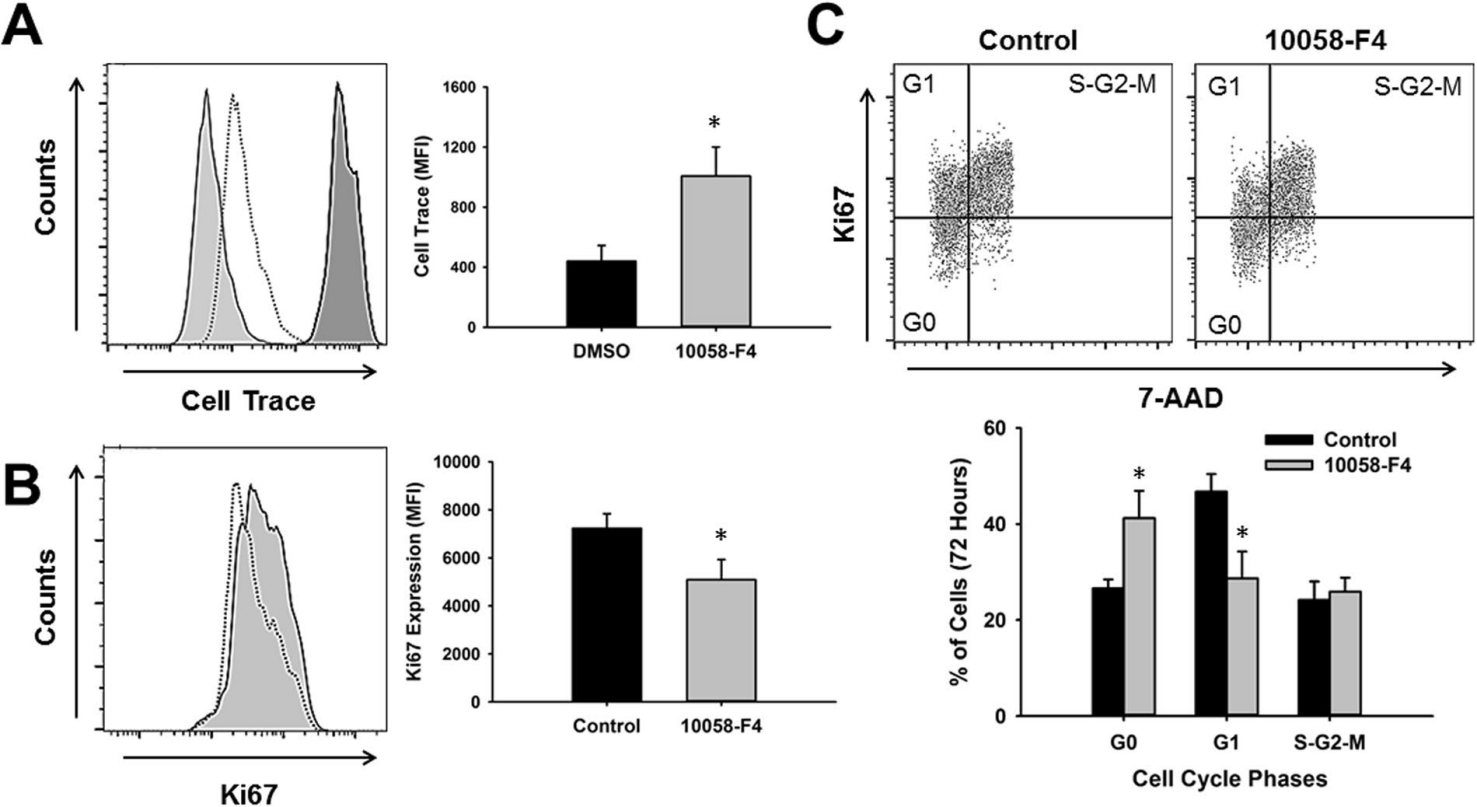
Inhibition of c-Myc decreases cardiac progenitor cell proliferation and induces quiescence. (**A**) Representative histogram showing CPC proliferation analysis by CellTrace dye dilution 72 hours after treatment with the c-Myc inhibitor 10058-F4 (dotted line) and DMSO control (solid line, light gray) compared to baseline (solid line, dark gray). Graph indicates the CellTrace mean fluorescence intensity showing less dye dilution in samples treated with the c-Myc inhibitor relative to DMSO control, an indication of delayed growth (n = 4, *p < 0.03). (**B**) Quantification of Ki67 expression 72 hours after treatment of CPCs with c-Myc inhibitor and DMSO control by fluorescence activated cell sorting. Graph indicates the average of Ki67 mean fluorescence intensity (MFI) in control (black bars) and c-Myc inhibitor-treated CPCs (gray bars) (n = 4, *p < 0.03). (**C**) Cell cycle analysis of CPCs after c-Myc inhibition. Dot Plots show representative images of cells stained with 7-AAD and Ki67 and analyzed 72 hours later. Graph indicates the average percentage of cells in each phase of the cell cycle in DMSO control (black bars) and c-Myc inhibitor treated CPCs (gray bars) (n = 4, *p < 0.05).

**Figure 6:**
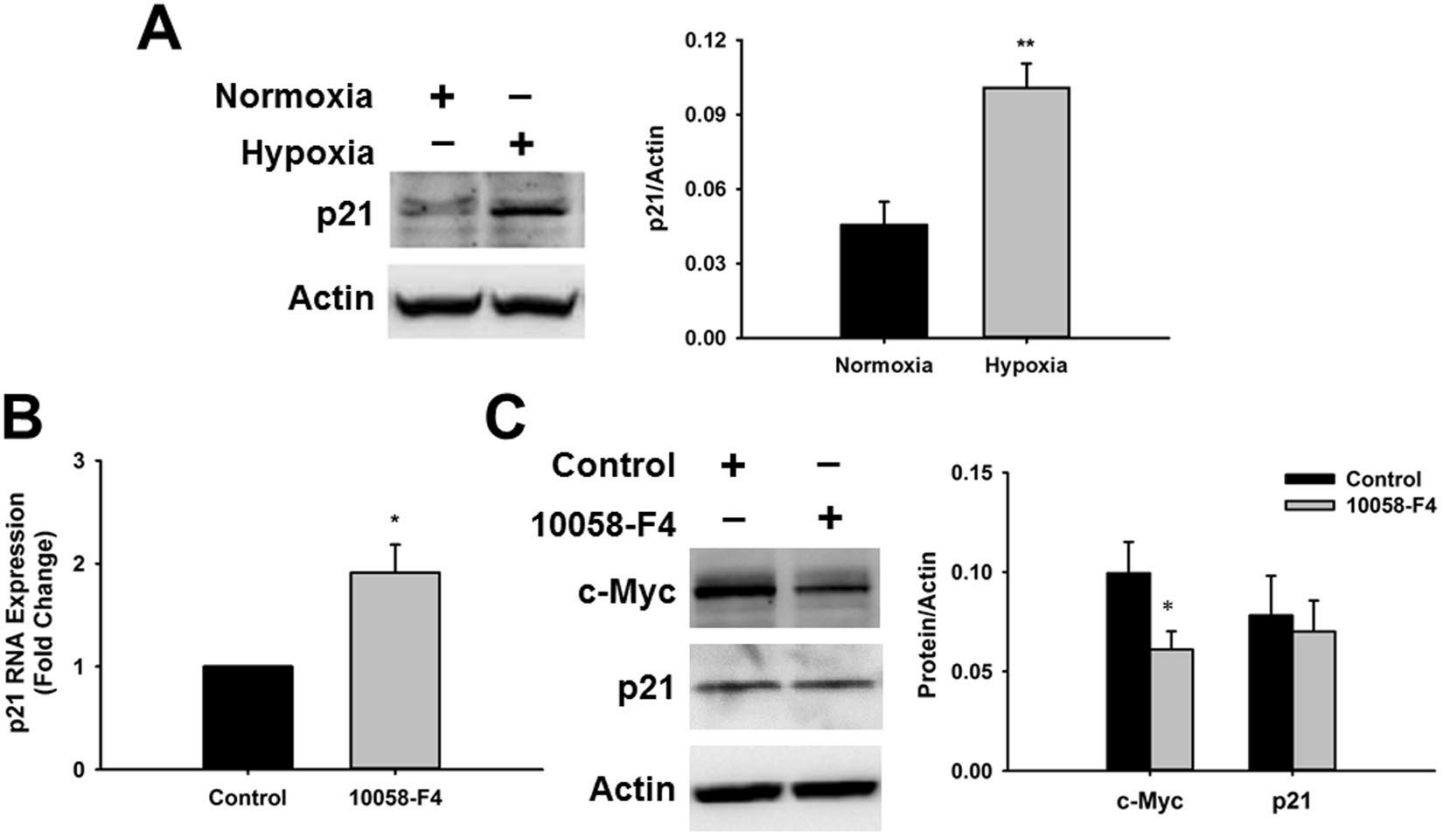
Hypoxic stress and c-Myc inhibition differentially affect the expression of the cell cycle inhibitor p21. (**A**) Representative western blot image showing induction of p21 in CPCs exposed to hypoxia relative to normoxia. Graph represents p21 expression determined by densitometry analysis of all blots normalized to Actin endogenous control (n = 5, **p < 0.006). (**B**) Real-time PCR analysis of p21 expression in CPCs treated with c-Myc inhibitor 10058-F4 for 24 hours relative to DMSO control (n = 5, *p < 0.03). (**C**) Representative western blot image showing the effect of c-Myc inhibition on c-Myc and p21 expression. Graph represents c-Myc and p21 expression determined by densitometry analysis of all blots normalized to Actin endogenous control (n = 4, *p < 0.03).

Treatment of CPCs for 72 hours with 10058-F4, a specific inhibitor of c-Myc that blocks its transcriptional activity, significantly reduced proliferation as demonstrated by a higher accumulation (143.10 ± 33.75%) of CellTrace proliferation dye in inhibitor-treated samples relative to vehicle control (1007.25 ± 194.02 vs 439.75 ± 104.93 mean fluorescence intensity, p < 0.02, n = 4) (Fig. 5A). Consistent with these results, we found that the expression of the cell cycle marker Ki67 was significantly reduced by 30.60 ± 8.30% (5090 ± 836 vs. 7226 ± 611 mean fluorescence intensity, p < 0.02, n = 4) after inhibition of c-Myc for the same length of time (Fig. 5B). To further confirm that c-Myc regulates CPC proliferation we performed cell cycle analysis. Our results show that 72 hours after c-Myc inhibition there is a significant increase in the number of cells accumulated in G0-phase relative to control by 53.6 ± 11.5% (41.22 ± 5.69 vs. 26.58 ± 1.87% of cells, p < 0.04, n = 4). This accumulation was associated with a decrease of 39.56 ± 9.1% in the number of cells in G1-phase relative to control (28.66 ± 5.56 vs. 46.72 ± 3.65% of cells, p < 0.02, n = 4) (Fig. 5D). Altogether, these findings support a role for c-Myc in the control of CPC proliferation.

c-Myc is known to control cell quiescence by direct targeting cell cycle proteins, among these, the cell cycle inhibitor p21^34^. We analyzed the expression of p21 under hypoxia and found that it was induced by 149.30 ± 41.33% (0.101 ± 0.009 vs. 0.046 ± 0.01 p21/Actin ratio, p < 0.005, n = 5) in CPCs exposed to hypoxia relative to normoxia (Fig. 6A). Therefore, we decided to investigate if p21 was also altered by c-Myc inhibition. Surprisingly, although p21 RNA expression was induced 1.91 ± 0.27-fold after inhibition of c-Myc (Fig. 6B), no changes were observed on p21 protein expression (Fig. 6C). To confirm the effectiveness of the inhibitor we analyzed the expression of c-Myc as it is known to regulate its own expression. As expected, c-Myc was significantly downregulated 40.12 ± 1.77% (0.061 ± 0.01 vs. 0.099 ± 0.016, c-Myc/Actin ratio, p < 0.03, n = 3) by inhibitor treatment.

### Hypoxic Stress Affects c-Myc Protein Stability

**Figure 7:**
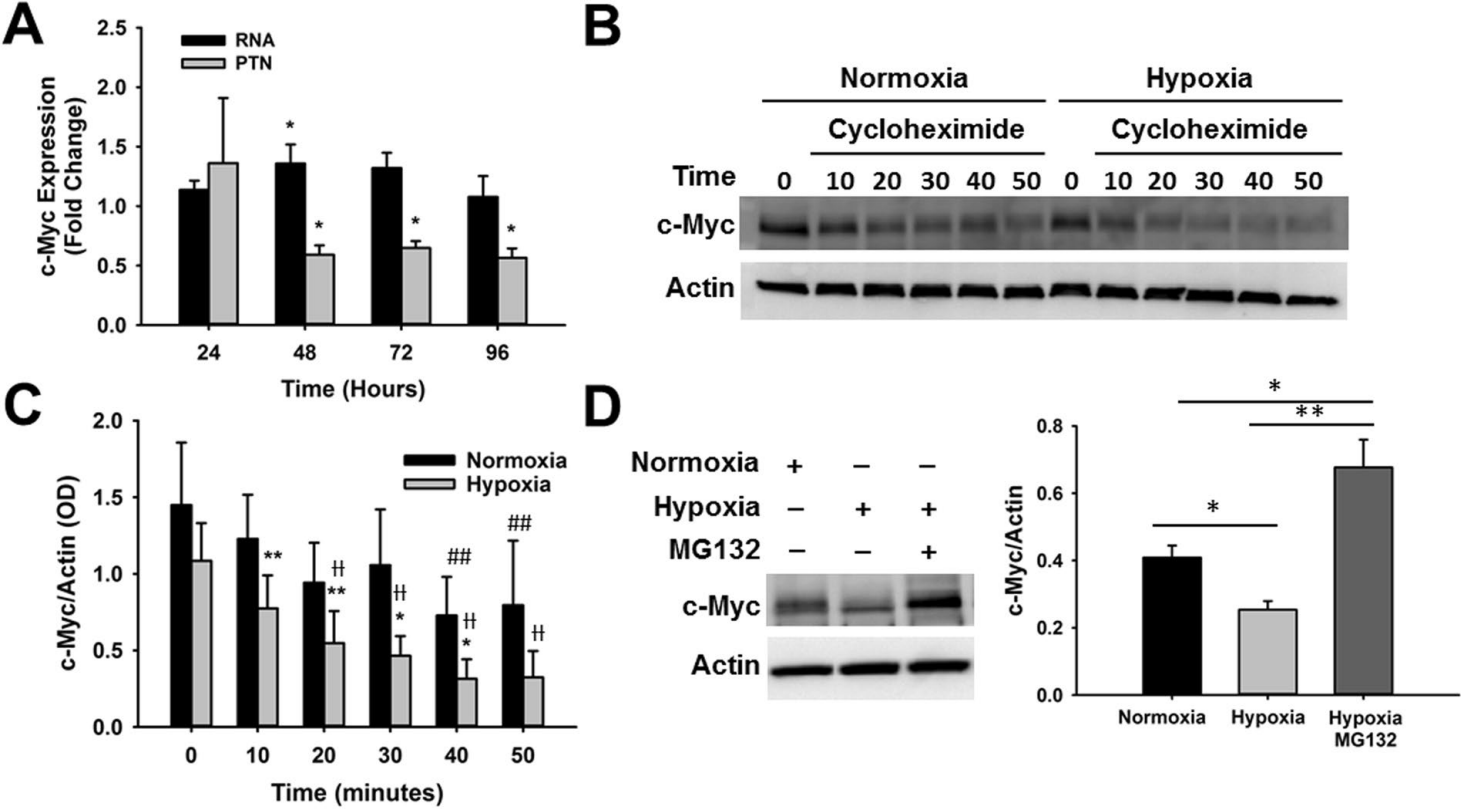
Hypoxic stress accelerates c-Myc protein degradation in cardiac progenitor cells. (**A**) Time-course analysis of c-Myc RNA (black bars) and protein (gray bars) expression under hypoxia relative to normoxia (n = 4–7, *p < 0.05). (**B**) Representative western blot image showing c-Myc protein degradation after translation blockage in CPCs cultured under normoxia and hypoxia for 48 hours. (**C**) Time-course analysis of c-Myc expression after protein synthesis blockage under normoxia (black bars) and hypoxia (gray bars) (n = 4–12, *p < 0.05, **p < 0.006, ^#^p < 0.005, ^Ɨ^p < 0.001). (**D**) Representative western blot image showing the effect of the proteasome inhibitor MG132 on c-Myc expression under hypoxia (n = 4, *p < 0.05, **p < 0.005). Graphs in C and D represent c-Myc expression determined by densitometry analysis of all blots normalized to Actin endogenous control. For statistical purposes, c-Myc expression was compared between groups for each time point (*) and between time points for each experimental group, normoxia control (#) and hypoxia (Ɨ).

The sustained decrease in c-Myc levels when CPCs are exposed to ischemic hypoxic stress suggests that a stable mechanism controlling c-Myc expression is activated under hypoxia. RNA analysis showed that reduction in c-Myc protein expression was not associated with changes in c-Myc RNA, suggesting that hypoxic stress affects c-Myc protein stability (Fig. 7A). To confirm this possibility, we analyzed c-Myc protein expression in CPCs cultured under normoxia and hypoxia for 48 hours after treatment with cycloheximide, an inhibitor of protein synthesis. Results show that in both normoxia and hypoxia c-Myc is rapidly degraded minutes after protein synthesis inhibition (Fig. 7B). However, under hypoxia, a significant decline in c-Myc expression occurred earlier than under normoxia, 20 min after protein blockage relative to baseline levels (0.55 ± 0.21 vs 1.09 ± 0.25, p < 0.001, n = 9) compared to 40 minutes (0.73 ± 0.25 vs 1.45 ± 0.41, p < 0.004, n = 4). The rate of protein degradation was higher under hypoxia, as indicated by reduction in c-Myc expression in all time points tested relative to normoxia, which was significant between 10–40 minutes. In just 10 minutes after protein synthesis blockage, c-Myc expression decreased 40.89 ± 8.37% in hypoxia relative to normoxia (1.23 ± 0.29 vs. 0.77 ± 0.22 c-Myc/Actin ratio, p < 0.006, n = 12). At the end of 40 minutes, c-Myc expression was down by 58.58 ± 2.79% (0.31 ± 0.13 vs. 0.73 ± 0.25 c-Myc/Actin ratio, p < 0.05, n = 4) (Fig. 7C). To further confirm that hypoxia was affecting c-Myc protein stability, CPCs were treated with the proteasome inhibitor MG132. As expected, 48 hours in hypoxia caused a decrease in c-Myc expression of 37.19% ± 6.37 (0.25 ± 0.03 vs. 0.41 ± 0.04 c-Myc/Actin ratio, p < 0.03 n = 4) (Fig. 7D). This decrease was significantly reversed by proteasome inhibition (0.68 ± 0.08 vs. 0.25 ± 0.03 c-Myc/Actin ratio, p < 0.001, n = 4) (Fig. 7D), further supporting our findings that c-Myc protein degradation is activated under hypoxic conditions.

### Reduction of c-Myc stability under hypoxia is independent of GSK-3β

**Figure 8:**
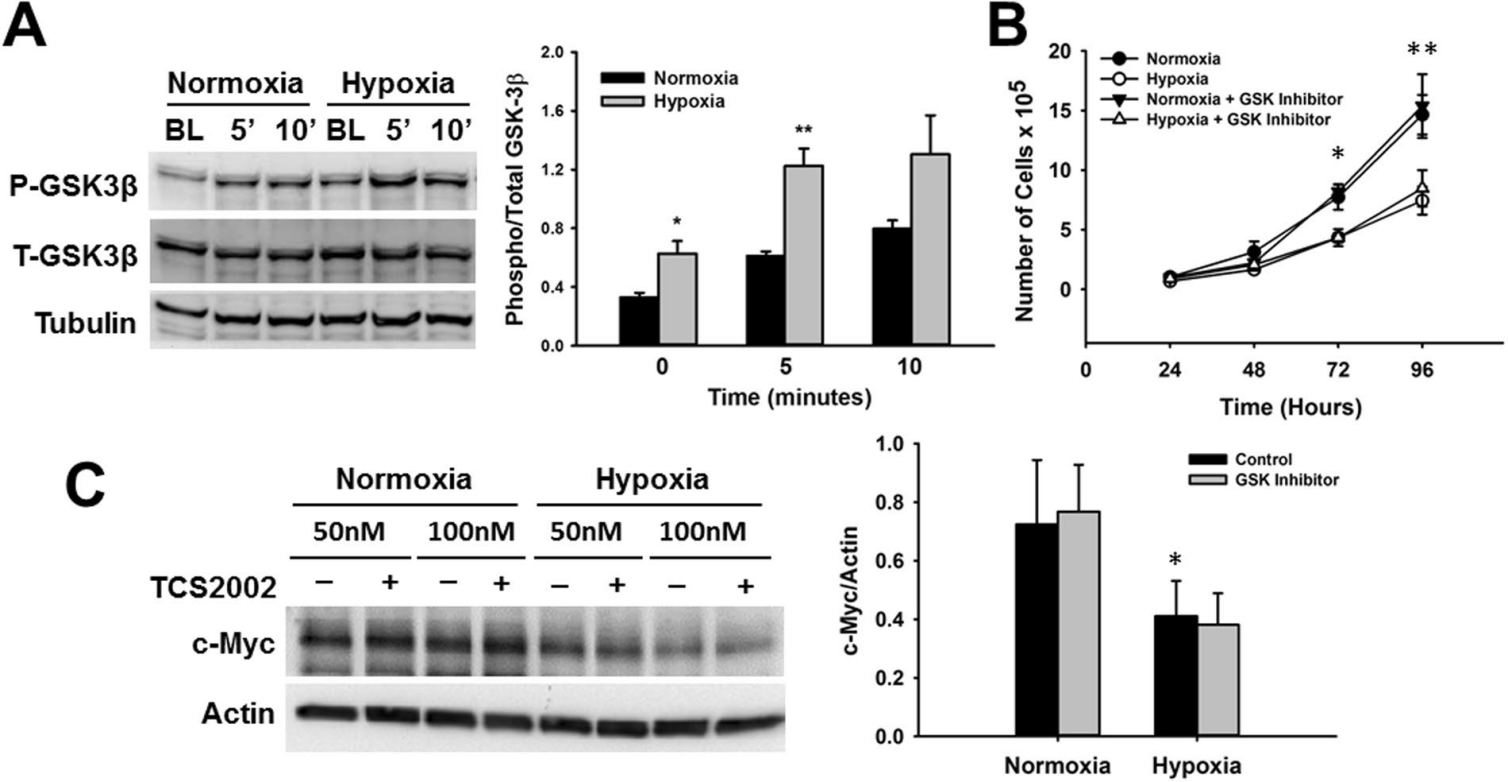
Expression of GSK-3β under hypoxia and effect on c-Myc expression. (**A**) Representative western blot image showing increased expression of inhibited GSK-3β (Ser9) under hypoxia (gray bars) relative to normoxia (black bars), after short stimulation with culture media. Graph represents Phospho/Total GSK-3β expression determined by densitometry analysis of all blots (n = 4, *p < 0.05, **p < 0.007). (**B**) Effect of GSK-3β inhibition by TCS2002 on CPCs proliferation under normoxia and hypoxia (n = 4, *p < 0.05, **p < 0.001). (**C**) Effect of GSK-3β inhibition by TCS2002 (gray bars) on c-Myc expression in CPCs cultured under normoxia and hypoxia relative to vehicle control (black bars). Inhibitor was tested at 50 and 100 nM. Graph represents c-Myc expression determined by densitometry analysis of all blots normalized to Actin endogenous control (n = 4, *p < 0.05).

GSK-3β is a major regulator of c-Myc protein stability that when activated promotes c-Myc degradation. Therefore, we investigated its role in c-Myc protein degradation under hypoxia. Surprisingly, we found that the expression of GSK-3β (Ser9), the inactive form of GSK-3β, was significantly increased under hypoxia (Fig. 8A), suggesting that although mechanisms to promote c-Myc stability were activated, increased degradation was still present. To confirm if GSK-3β has any impact on CPCs proliferation and c-Myc expression, we cultured CPCs in the presence of the GSK-3β inhibitor TCS2002. As expected, hypoxia caused a reduction in CPCs proliferation (n = 4, *p < 0.05, **p < 0.005) in association with a decrease in c-Myc expression relative to normoxia (n = 3, *p < 0.05). However, inhibition of GSK-3β had no effect on CPC growth in either normoxia or hypoxia (Fig. 8B). Likewise, GSK-3β inhibition had no impact on c-Myc expression under either condition (Fig. 8C). These findings suggest that GSK-3β is not involved in the control of CPC growth or changes in c-Myc expression under stress conditions such as hypoxia or under normal growth.

## Discussion

The goal of our study was to identify possible mechanisms that lead to impairment of CPC regenerative potential under ischemic stress. We provide evidence that ischemic hypoxia drives CPCs into a quiescence state, limiting both self-renewal and vasculogenic potential. These findings were associated with downregulation of the proto-oncogene c-Myc and upregulation of the cyclin inhibitor p21.

The adult stem cell niche works to preserve the balance of self-renewing, uncommitted progenitor cells with differentiated cells. This balance is achieved by the delicate regulation of both intrinsic and extrinsic factors. Oxygen concentration is a critical component of niche biology. Oxygen gradients are often found in stem cell niches, where hypoxic regions help to preserve slowly dividing and undifferentiated immature cells^49–52^. How cells respond to hypoxia largely depends on oxygen concentration, duration of exposure and cell type. Our group has previously shown that changes in oxygen concentration play an important role in human cardiac stem cell proliferation and migration^33^. Mild hypoxic conditions in the range of 3–5% O_2_ have been shown to exert beneficial effects on different types of stem cells. Bone marrow-derived stromal cells grown under 3% O_2_ show increased self-renewal and significant decrease in differentiation, apoptosis and senescence^53^. Similarly, hypoxia maintains neural stem cells in an undifferentiated state and promotes survival and proliferation^54–56^. Such positive functional responses steered several studies on the use of hypoxia as a pre-conditioning strategy prior to cell transplantation to enhance therapeutic potential in experimental ischemia and other injury models^57–60^. Regarding CPCs, some studies have shown that mild-hypoxia (5% O_2_) confers improvement in proliferative capacity and vasculogenic potential^61^, as well as preservation of genomic stability through maintenance of redox balance^62^.

In the case of severe hypoxia (0.5–1% O_2_), studies are controversial. Extreme hypoxia mimicking ischemic injury as we tested has been used for pre-conditioning of CPCs prior to transplantation and shown beneficial effects^58^. Our results indicate that exposure to ischemic hypoxia (0.5% O_2_) severely impacted CPC proliferation and vasculogenic potential. In support of our findings, studies on MSCs have shown that 0.5% hypoxia also has a negative impact by triggering apoptosis^63^. The key factors that drive these differences are likely to be both timing and degree of hypoxia. In fact, the positive effect of 0.5% hypoxia on CPC function was time dependent, and when extended to more than 12 hours caused adverse effects leading to cell death. This result is further supported by a report showing potential benefits of short-term exposure of bone-marrow derived progenitor cells to 1% hypoxia, which were reversed by prolonged hypoxia^64^, similar to our findings. One of the main differences we observed in our study relative to others is that, despite reports showing an increase in cell death after long-term exposure to ischemic hypoxia, we did not find any evidence of alterations in cell survival.

Most cell types respond to stress by undergoing apoptosis, development of senescence or quiescence. We found that exposure of CPCs to hypoxic stress reduces cell proliferation by promoting quiescence. Impairment of CPC proliferation by hypoxia was not associated with development of senescence, and was in fact reversible, as CPCs placed back into normoxic conditions after exposure to ischemic hypoxia are able to recover their growth rate. This finding is supported by studies linking hypoxia to preservation of stem cell homeostasis and self-renewal^51, 65^. Although one of the main purposes of quiescence for adult stem cells is to preserve stemness, imbalance in the mechanisms involved in its regulation may compromise tissue regeneration^66^. Different from embryonic stem cells, due to their limited self-renewal potential, adult stem cells must develop mechanisms that allow rapid transition between quiescence and cell cycle re-entry. These mechanisms are essential for maintenance of stem cell niches within different tissues throughout life and response to regeneration needs after injury, while avoiding malignant transformation^67^. Adult stem cell quiescence is regulated by transcription factors that play an important role by targeting genes involved in the control of cell cycle progression^68–70^. In our work, we provide evidence that the expression of c-Myc is downregulated during hypoxia-induced CPC quiescence. The involvement of c-Myc in the regulation of CPC growth/quiescence state is further supported by our findings showing a significant effect of c-Myc inhibition on CPC proliferation and cell cycle progression. Regulation of hematopoietic stem cell quiescence by the transcription factor GATA-2 has been shown to depend on c-Myc control of cell cycle inhibitors such p21^71^. The cell division control protein Cdc42 regulates hematopoietic stem cell quiescence through modulation of c-Myc and the cyclin-dependent kinase inhibitor p21^72^. Outside of the hematopoietic niche, down-regulation of c-Myc and up-regulation of p21 also correlate with quiescence of pancreatic progenitor cells^73^. Our results show that p21 is upregulated under hypoxia, while c-Myc is downregulated. Surprisingly, although c-Myc inhibition induces the expression of p21 transcripts, it had no impact on p21 protein expression. These findings are supported by reports showing that p21 is a transcriptional target of c-Myc^74^. Furthermore, they bring other potential regulatory steps to the hypoxia scenario that may affect p21 protein expression independent of c-Myc. Our experiments are limited in this context, as it is unlikely that c-Myc inhibition alone will exactly image what is observed under hypoxia. For example, we have found that the expression of the histone deacetylase Sirt1 is also downregulated under hypoxia and plays a role in CPCs self-renewal (Supplementary Figure 3). Likewise, downregulation of Sirt1 in mesenchymal stem cells has been shown to inhibit proliferation and this effect was associated with upregulation of cyclin inhibitors p21 and p16^75^. Independent of the context, similar to our findings, most studies in the literature indicate that the control of cellular quiescence by c-Myc is associated with upregulation of p21 expression. Considering p21 as an endpoint, impairment of p21 function by deletion or knockdown has been shown to be sufficient to block quiescence, and high p21 levels are necessary not only to enter, but also maintain a quiescence state in different experimental models^76^. Although we have focused our work on the analysis of c-Myc and p21 expression, as a central regulator of proliferation, c-Myc is expected to target the cell cycle at many different steps. Therefore, we cannot discard an effect on several other proteins required for CPC cell cycle progression under hypoxia.

c-Myc has been recognized as a stress sensor^77^, and its expression is altered in different injury models, including acute myocardial infarction^78–82^. Under normal physiological conditions, c-Myc expression is tightly regulated by concerted action of extracellular kinase 1/2 (ERK) or c-JUN N-terminal kinase (JNK), and glycogen synthase kinase-3β (GSK-3β) activity, through AKT-mediated phosphorylation^83, 84^. We found that hypoxia-induced downregulation of c-Myc in CPCs is mediated by mechanisms that involve protein degradation. Interestingly, although we observed activation of AKT pathway (Supplementary Figure 4) in our study and expected increase in the inhibited form of GSK-3β, indicating that mechanisms favoring stabilization of c-Myc were active, degradation was still evident. These findings suggest that other mechanisms involved in the control of c-Myc stability are affected in CPCs by hypoxic stress. Regulation of c-Myc levels by hypoxia could be affected during late steps of the protein degradation cascade involving ubiquitination as demonstrated for other stem cell types^85–88^. Alternatively, AKT has been shown to activate pathways that regulate GSK-3β-independent c-Myc degradation^89^ and GSK-3β-dependent stabilization of p21^90^. Although currently we do not have an explanation for altered kinase activities under hypoxia, activation of pro-survival mechanisms involving AKT is a common effect of hypoxia on different types of stem/progenitor cells, indicating an essential role of this pathway^53, 57, 64^.

## Conclusions

Although CPCs have demonstrated beneficial regenerative effects in experimental models, insufficient myocardial repair under conditions of severe hypoxia such as ischemic cardiomyopathy suggests that their regenerative capacity *in situ* is limited. Identifying the molecular control switch that regulates hypoxia-induced quiescence in CPCs is essential when considering therapeutic approaches designed to stimulate endogenous cardiac regenerative potential in ischemic disease. The goal of the present study was to identify mechanisms that regulate CPC functions required for regeneration under ischemic hypoxia. Our work provides novel insights into the effect of hypoxia on the induction of CPC quiescence. Importantly, it suggests that hypoxia-induced deregulation of c-Myc expression and promotion of quiescence, even as a protective mechanism, may affect cardiac regeneration through impairment of essential cellular functions required for progenitor-induced tissue repair under ischemic injury.

### Significance

Stem cell therapy is an emerging approach for the treatment of heart failure. The adult myocardium contains reservoirs of cardiac progenitor cells (CPCs) capable of promoting revascularization and myocardial regeneration. However, ischemic stress impairs their regenerative potential. Identification of molecular mechanisms that impair CPC ability to promote regeneration is vital for therapeutic applications. Here we show that ischemic stress induces CPC quiescence while we also identify c-Myc as a novel regulator of ischemia-induced CPC functional impairment. Our findings provide significant insights into how hypoxic stress regulates essential progenitor functions required for myocardial regeneration.

## Electronic supplementary material

Supplementary Information
