# Supplementary material for: Hypoxic Stress Decreases c-Myc Protein Stability in Cardiac Progenitor Cells Inducing Quiescence and Compromising Their Proliferative and Vasculogenic Potential
Source: Sci Rep. 2017 Aug 29;7:9702. doi: 10.1038/s41598-017-09813-x (PMC5575078; doi:10.1038/s41598-017-09813-x)
Supplement: Supplementary file 1 — Supplementary Information [file 41598_2017_9813_MOESM1_ESM.pdf]

Supplementary Information

Hypoxic Stress Decreases c-Myc Protein Stability in Cardiac Progenitor Cells Inducing Quiescence and Compromising Their Proliferative and Vasculogenic Potential

Michael A. Bellio[1#], Mariana T. Pinto[1#], Victoria Florea[1#], Paola A. Barrios[1], Christy N. Taylor[1], Ariel B. Brown[1], Courtney Lamondin[1], Joshua M. Hare[1,2], Ivonne H. Schulman[1,3] and Claudia O. Rodrigues[1,4]*

Supplementary Figures Legends

Supplementary Figure 1. Effect of 5% hypoxia in cardiac progenitor cell proliferation. **(A)** Time-course analysis of CPC growth represented by number of cells under normoxia (closed circles) and hypoxia (open circles) over a total period of 96 hours (n = 4).

Supplementary Figure 2. Time course analysis of c-Myc protein expression under hypoxia relative to normoxia. Representative western blot images showing a decrease in c-Myc protein expression under hypoxia at different time points for several experiments. Graph represents c-Myc expression determined by densitometry analysis of all blots normalized to Actin endogenous control (n = 4-6, *p<0.05).

Supplementary Figure 3. Effect of hypoxia on Sirt1 expression and Sirt1 knockdown on CPCs proliferation. **(A)** Representative western blot image showing a time-dependent decrease in Sirt1 after exposure of CPCs to hypoxia. Graph represents Sirt1 expression determined by densitometry analysis of all blots normalized to Actin endogenous control (n = 4-6, *p<0.05, ***p<0.0005). **(B)** Representative western blot image showing lentiviral mediated Sirt1 knockdown

in CPCs. Graph represents Sirt1 expression determined by densitometry analysis of all blots normalized to Actin endogenous control (n = 3, *p<0.05) **(C)** Effect of Sirt1 knockdown on CPCs proliferation assessed 72 hours after BrdU incorporation (n = 3,*p<0.05).

Supplementary Figure 4. (A) Representative western blot image showing time-course analysis of phospho-AKT (Ser473) expression after exposure of CPCs to hypoxia. Graph represents P-AKT expression determined by densitometry analysis of all blots normalized to Gapdh endogenous control (n = 3). **(B)** Representative western blot image showing increased expression of phospho-AKT (Ser473) under hypoxia relative to normoxia, after short stimulation with culture media (n = 7). Graph represents Phospho/Total-AKT expression determined by densitometry analysis of all blots (n = 4, *p<0.05).

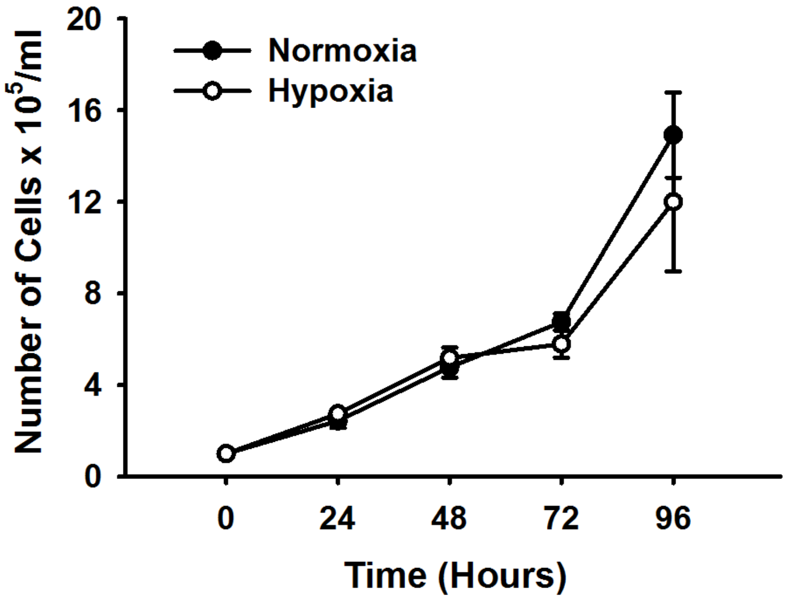

Supplementary Figure 1

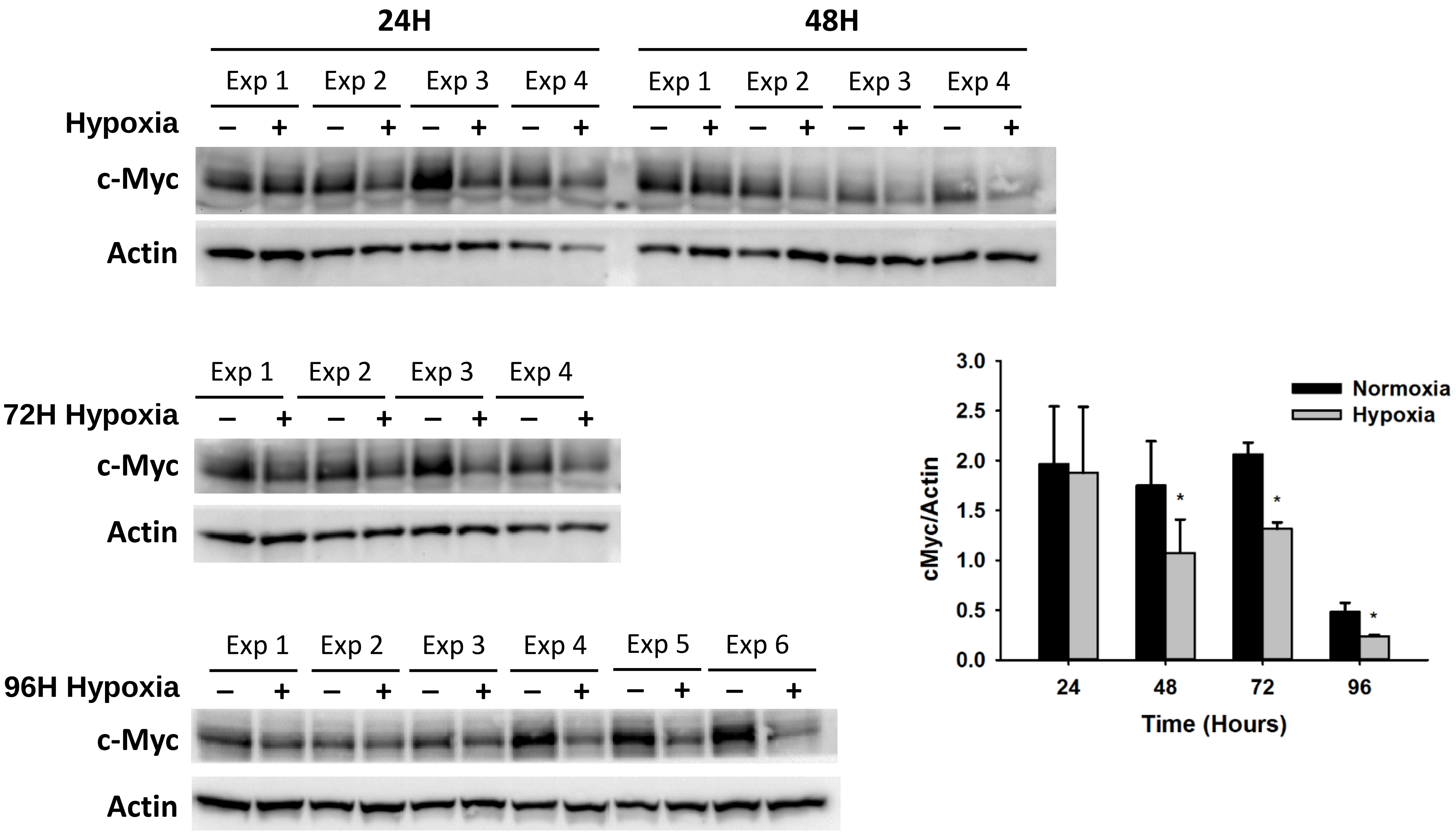

Supplementary Figure 2

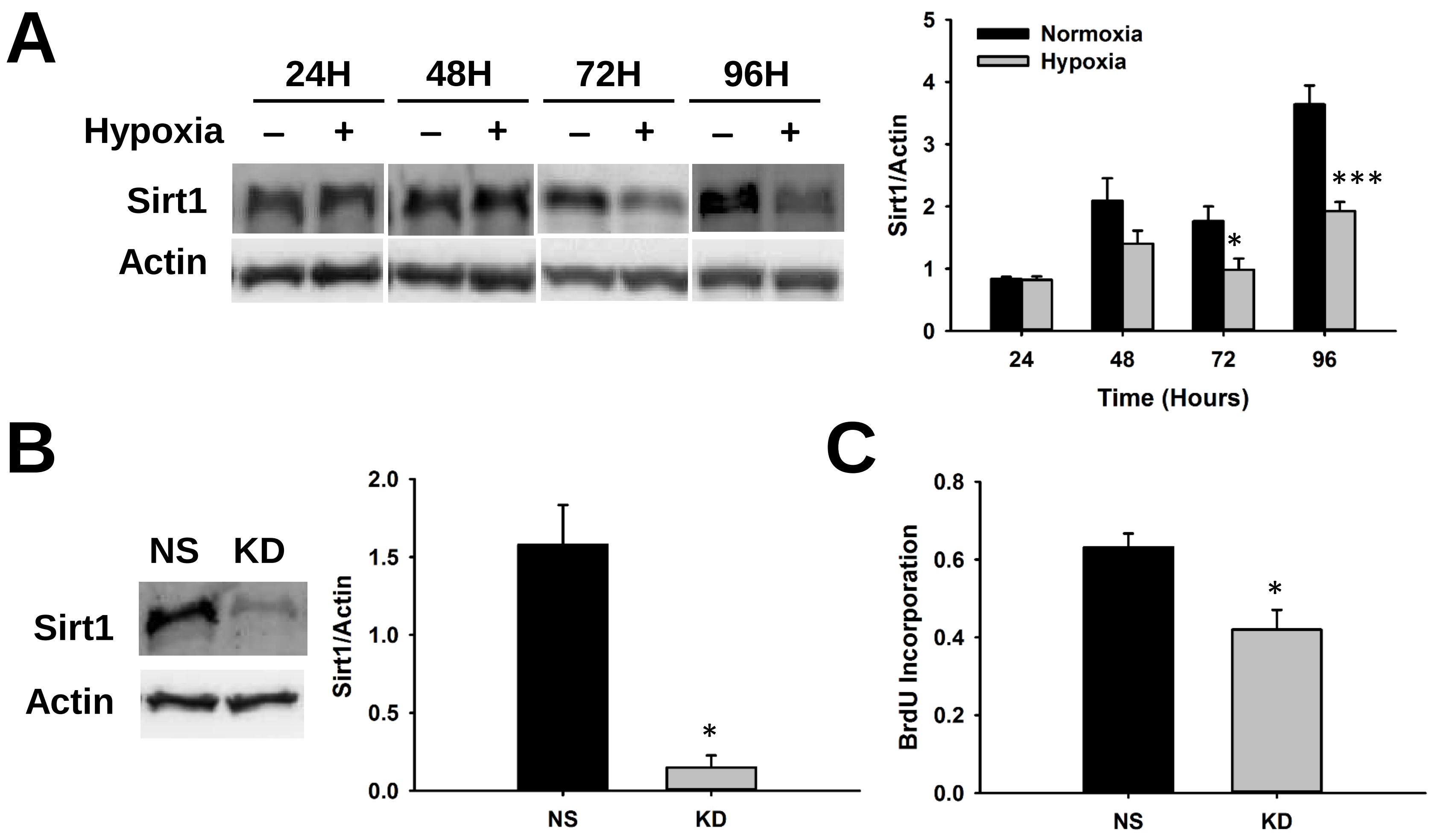

Supplementary Figure 3

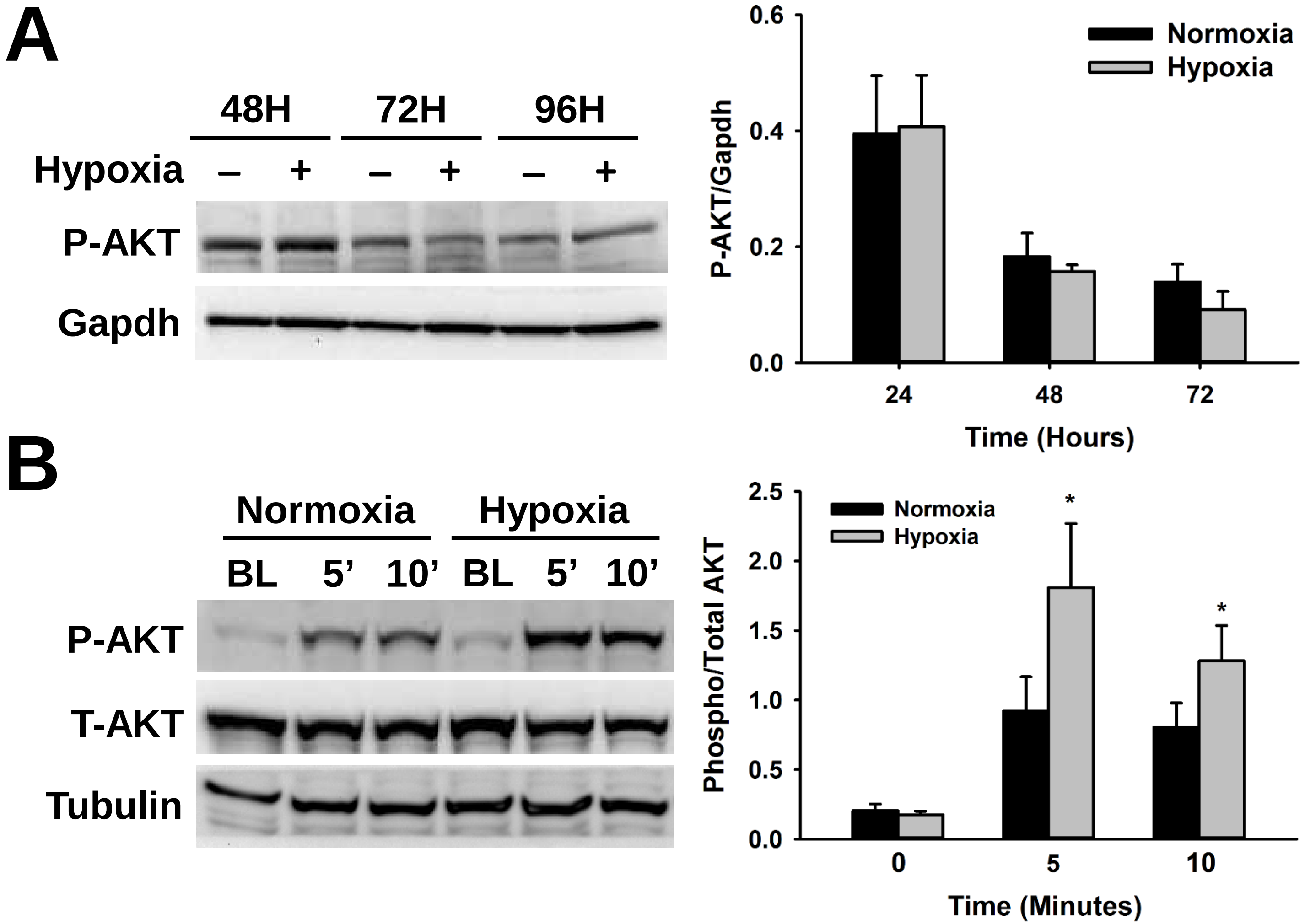

Supplementary Figure 4
